# Naturally‐occurring dietary salicylates in the genesis of functional gastrointestinal symptoms in patients with irritable bowel syndrome: Pilot study

**DOI:** 10.1002/jgh3.12578

**Published:** 2021-07-21

**Authors:** Caroline J Tuck, Sreepurna Malakar, Jacqueline S Barrett, Jane G Muir, Peter R Gibson

**Affiliations:** ^1^ Department of Gastroenterology Alfred Hospital and Monash University Melbourne Victoria Australia; ^2^ Present address: La Trobe University Melbourne Victoria Australia; ^3^ Present address: Chemwatch Melbourne Victoria Australia; ^4^ Present address: Diet Solutions Melbourne Victoria Australia

**Keywords:** diet therapy, irritable bowel syndrome, salicylates

## Abstract

**Background and Aim:**

An elimination‐rechallenge dietary approach targeting naturally‐occurring bioactive chemicals has been proposed to alleviate functional gastrointestinal symptoms. A major focus of this approach is salicylates. This study aimed to address the potential role of dietary salicylates in the induction of symptoms in patients with irritable bowel syndrome (IBS).

**Methods:**

A pilot, double‐blind, randomized, cross‐over trial of 2‐week low‐ *versus* high‐salicylate diets (6.6 and 27.9 g/day salicylate, respectively) was undertaken. All foods were provided containing minimal quantities of other potential food triggers. Gastrointestinal and extraintestinal symptoms were measured daily using a 100‐mm visual‐analogue‐scale.

**Results:**

Ten participants with IBS completed the study, including one with known aspirin‐sensitivity. Overall, no differences in symptoms were observed (*P* = 0.625; Friedman test). However, clear symptom provocation was seen in the aspirin‐sensitive participant, with all abdominal symptoms and tiredness worsening during the high‐salicylate diet. A similar trend was seen in another participant, where abdominal symptoms gradually worsened during the high‐salicylate diet.

**Conclusions:**

These results provide some evidence that food‐related salicylates may influence the genesis of symptoms in a subset of patients with IBS. A larger cohort is needed to determine the incidence of salicylate‐sensitivity and further evaluate the diet as a potential therapeutic target.

The protocol was registered at www.anzctr.org.au (ACTRN12620001250921).

## Graphical abstract

This pilot randomised controlled trial (RCT) provides valuable data regarding the potential use of a diet low in naturally‐occurring dietary salicylates to reduce symptoms associated with common conditions such as irritable bowel syndrome.

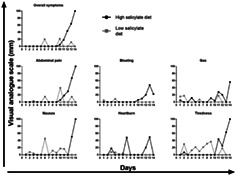

## Introduction

Worldwide interest in the use of diet as a therapeutic tool in patients with irritable bowel syndrome (IBS) has grown. This attention has been stimulated by the well‐documented induction of symptoms by the ingestion of specific foods,^1^ patient interest in using diet as a self‐management tool, and the considerable evidence‐base for the efficacy of the low fermentable oligo‐, di‐, mono‐saccharides and polyols (FODMAP) diet.^2^, ^3^

An elimination‐rechallenge dietary approach developed by the Royal Prince Alfred Hospital Allergy Clinic in Sydney^4^ targets bioactive chemicals in food and follows a process that is largely empirical and based on clinical observation. In the elimination phase of this diet, predominantly naturally‐occurring chemicals in food like salicylates, amines, and glutamates are highly restricted in the patient's diet. Once the symptoms are controlled, the patients are re‐challenged with individual foods high in particular chemicals to detect specific food chemical hypersensitivities so that longer‐term management strategies can be formulated. It has been argued that symptoms occur due to gradual accumulation over many days of food chemicals in tissues in a dose‐dependent manner, and hence decreasing the amount and frequency of food chemicals in the diet may alleviate symptoms.^5^ Mechanisms for symptom induction are not determined, but might include activation of mast cells and COX‐1‐dependent inflammatory pathways.^6^ Unfortunately, there is limited critical study of this dietary approach. Good symptom control has been reported,^7^ but problems of palatability^4^ and nutritional adequacy in children^8^ have been reported.

Of the multitude of chemicals that are restricted with this approach, salicylates are believed to be a major contributor to symptoms. Thus, in 78% of patients with a range of chronic symptoms who responded to food‐chemical restriction, re‐challenge with salicylates was associated with symptom recurrence,^7^ 12% of patients with IBS reported symptoms resulting from the use of nonsteroidal anti‐inflammatory drugs (NSAIDs),^9^ and high dose aspirin was reported to be associated with bloating in smokers.^10^ The best documented cases of salicylate intolerance include the classic symptom triad manifesting urticaria, angioedema, and nonallergic asthma^11^ and in aspirin‐exacerbated respiratory disease.^12^ Whether such sensitivity relates to ingestion of medicinal aspirin only or also relates to food‐associated salicylates, which have structural differences to and are consumed in lower amounts, remains poorly studied. Salicylates are present widely in plants and are bioavailable. Salicylates are detected in serum and urine of subjects not taking aspirin,^13^ and higher serum levels are detected in vegetarians compared with nonvegetarians.^14^

The present study aimed to address the potential role of salicylates in the induction of functional gastrointestinal symptoms by performing a pilot, double‐blind, randomized, cross‐over trial of low‐ *versus* high‐salicylate diets in IBS patients.

## Methods

### Participants

Patients with IBS based on Rome III criteria^15^ were recruited between March 2014 and May 2015 via advertisements on the website of the Department of Gastroenterology, Monash University and private dietitian clinics. Exclusion criteria comprised coeliac disease, pregnancy, and breastfeeding, and other significant comorbid conditions such as diabetes and inflammatory bowel disease. They must not have had previously received any advice or information on a low chemical diet. Participants were not permitted to take pharmacological agents like laxatives to alter their symptoms. They could not be on aspirin or any other drugs containing or delivering salicylates for at least 2 weeks prior to study commencement.

### Study protocol

Potential participants were screened by a gastroenterologist to confirm the diagnosis and to classify according to their predominant bowel habit into diarrhea (IBS‐D), constipation (IBS‐C), or mixed type (IBS‐M). During the 7‐day baseline data‐collection period, all participants were required to record their habitual dietary intake in a detailed food diary. They also recorded symptoms each day during those 7 days. Semiquantitative analysis of their baseline food intake for salicylate and FODMAP contents was performed by two independent dietitians.

Participants were then randomized by a computer‐generated order to receive a diet either high or a low in salicylate content for the next 14 days. Participants and investigators were blinded to the diet. After the initial 14 days, the participants undertook a washout period of 7 days, where they resumed their habitual diet. During the washout phase, they completed a diary recording their dietary intake and symptoms. This was followed by a cross‐over to the alternate diet for further 14 days. During the interventional dietary phases, all foods were provided and participants recorded the quantity of the provided foods consumed along with their symptom scores.

Written, informed consent was obtained from all participants prior to commencement of the study. The study protocol was approved by Monash University Human Research Ethics Committee, Monash University, Clayton (Project number: CF14/1642 – 2014000780). The protocol was registered retrospectively at www.anzctr.org.au (ACTRN12620001250921).

### Interventional diets

All foods including three main meals, morning and afternoon snacks, and drinks were provided (Table 1). Detailed meal plans for high‐ and low‐salicylate diets were provided. For some meals that included fresh salads, detailed recipes were provided for participants to prepare before consumption. They were strictly instructed to avoid eating out during the study. If participants wanted to eat foods that were not specified on the supplied list, they contacted the study investigator for guidance on food choices.

**Table 1:** Sample meal plan of low‐ and high‐salicylate diets provided

	Low‐salicylate diet example day	High‐salicylate diet example day
Breakfast	Porridge made with half cup quinoa, half cup lactose‐free milk, three‐fourths cup blueberries, 1 tsp. maple syrup. 1 cup of English breakfast tea.	Porridge made with half cup quinoa, half cup lactose‐free milk, one‐third cup pineapple†, half cup strawberry†, half cup kiwi fruit†, 1 tsp. maple syrup. 1 cup of English breakfast tea.
Snack	1 slice lemon syrup cake. 1 cup of English breakfast tea.	Strawberry smoothie†. 1 cup of English breakfast tea.
Lunch	Asian soup (containing choy sum, cabbage, beansprouts, sugar snap peas, ginger, chives, pepper, lemon grass) with 1 slice panini bread.	Asian soup (containing corn flour, bok choy, pumpkin squash†, cumin†, green beans†, ginger†, chives†, pepper, lemon grass) with 1 slice panini bread.
Snack	140 ml lactose free yoghurt with half cup blueberries.	140 ml lactose free yoghurt with one‐fourth cup kiwi fruit†, and one‐third cup cantaloupe†.
Dinner	Lamb kebab with salad containing 2 cups iceberg lettuce, half cup cucumber, 4 asparagus, 1 cup endive, 2 tsp. lemon juice, 1 tsp. olive oil, salt, pepper.	Lamb kebab with salad containing 2 cups baby cos lettuce†, half cup cucumber†, one‐fourth cup white radish†, 2 tsp. lemon juice†, 1 tsp. olive oil, salt, pepper.

^†^ Denotes foods with higher salicylate content as per Malakar et al. 2017.^16^

The study investigator and two professional chefs prepared all foods. Frozen, complete meals were provided to the participants with instructions to thaw and heat before consumption. The foods were delivered biweekly free of charge to the participant homes. Compliance was monitored via the daily food diaries.

The diets were designed based upon the salicylate content of foods determined by in‐house analysis using GC–MS.^16^ Both diets were free of gluten, preservatives, additives and lactose, were consistent in their minimal content of FODMAPs, and contained moderate levels of amines and glutamates as per published food content^4^, ^17^, ^18^, ^19^, ^20^, ^21^, ^22^, ^23^, ^24^, ^25^, ^26^, ^27^, ^28^, ^29^, ^30^, ^31^ to avoid any confounding factors that might affect symptoms. The average daily intake of salicylates in the low‐salicylate diet was approximately 6 mg, and for the high‐salicylate diet, it was 28 mg. The meal plans had an average energy value of 8 MJ daily and met the recommended serves of all food groups according to the Australian dietary guidelines.^32^

Both the high‐ and low‐salicylate diets were analyzed for content of energy, macronutrients, fiber, and FODMAPs via FoodWorks 7 (Xyris Software Pty Ltd.; Brisbane, Qld, Australia). Data on FODMAP content were based on the Monash University FODMAP food composition database.

### Gastrointestinal and non‐gastrointestinal related symptoms

Gastrointestinal and non‐gastrointestinal symptoms were measured daily during the entire course of the study. Abdominal symptoms were measured using the visual analogue scale (VAS), while the fecal symptoms were measured using a Likert scale. On a 100 ‐mm VAS scale, 0 indicates complete remission of symptoms and 100 mm represents worst symptoms. Overall gastrointestinal symptoms, abdominal pain, bloating, passage of wind, and nausea were assessed using the VAS as previously applied.^33^ The number of bowel movements per day was recorded; 3 or more bowel actions were recorded as “3.” Urgency, straining, and sense of complete evacuation were measured using a three‐point Likert scale, where 0 indicated never, 1 indicated sometimes, and 2 indicated always. Fecal form was rated using the Bristol Stool Scale that classifies stools into seven categories. Extraintestinal symptoms, headache/migraine, rhinitis (stuffy or runny nose, post‐nasal drip), asthma, eczema, urticaria, and tiredness were also recorded using a 100‐mm VAS.

### Endpoints

The primary endpoint was the difference in overall gastrointestinal symptoms measured by the 100‐mm VAS on the low‐salicylate diet compared with high‐salicylate diet averaged over the last 3 days of each of the interventional dietary periods. Secondary endpoints included differences between the diets in specific abdominal and extraintestinal symptoms, and differences in symptoms between interventional diets and their respective preceding control phases of baseline or washout. All comparisons were made using average scores of the last 3 days of the respective dietary periods.

### Blinding

Both participants and researchers were blinded to the dietary treatments. To assess the success of blinding, at completion each participant was asked to identify their perception of the nature of the diets. As for the researcher, the diets were given a code and it was not revealed until data entry was locked.

### Statistical analysis

This was a pilot study and hence power calculations were not undertaken. Data were analyzed on an intention‐to‐treat basis. Parametric and nonparametric analyses were used as appropriate. For nonparametric analyses, Friedman test and Wilcoxon signed rank tests were performed to compare the abdominal and fecal symptoms between high‐ and low‐salicylate dietary interventions. *P* value of ≤0.05 was considered statistically significant. Statistical analysis was performed with GraphPad Prism (GraphPad Software, Version 6, La Jolla, CA, USA) program.

## Results

### Participants

The participant recruitment flowchart is shown in Figure S1. Baseline characteristics of the 10 participants studied are detailed in Table 2. One female participant had previously been diagnosed with aspirin‐sensitivity manifested by intermittent bouts of urticaria. However, urticaria was not present during recruitment and she had no previous engagement with a dietitian to avoid food‐related salicylates. Only two participants had seen a dietitian and both had not responded to a low FODMAP diet. Neither had been instructed on a low chemical diet. All 10 participants completed the study as per protocol.

**Table 2:** Demographics and clinical characteristics of the participants

				Content of habitual diet†		Baseline symptoms score (VAS) (mm)‡					
Age (years), sex	BMI (kg/m^2^)	Diagnosis	Prior dietary interventions	Salicylates	FODMAPs§	Overall	Pain	Bloating	Wind	Nausea	Heartburn
29, F	23.4	IBS–M	None	Moderate	Moderate	3.2	2.3	2.6	2.9	47.5	40.4
26, F	23.4	IBS‐D	Failed low FODMAP	High	High	28.2	32	36.2	19.1	25.9	17.5
67, F	25.2	IBS‐C, urticaria¶	None	Low	Moderate	0.0	0.0	0.0	8.4	0.0	0.0
70, F	33.7	IBS‐D	None	High	High	5.4	3.6	4.2	10.9	1.0	1.0
43, M	30.1	IBS‐M	None	High	High	18.8	20.7	26.2	27.2	0.0	20.1
45, F	38.2	IBS‐M	None	Moderate	Low	3.2	2.9	6.2	4.2	0.0	0.0
55, F	21.3	IBS‐M	None	Moderate	High	0.8	0.7	0.6	0.5	0.2	0.1
32, M	27.8	IBS‐M	None	Moderate	High	50.5	55.7	44.3	53.7	3.6	2.6
22, F	22.8	IBS‐C	Failed low FODMAP	High	Low	49.5	35.9	55.7	33.7	36.6	0.0
67, F	25.1	IBS‐C	None	High	High	3.4	2.5	2.9	5.9	0.8	1.1

^†^ Semiquantitative analysis of baseline food intake for salicylate and FODMAP contents were performed by two independent dietitians.

^‡^ Mean scores from day 5 to 7 according to the VAS.

^§^ FODMAP denotes fermentable oligo‐, di‐, mono‐saccharides and polyols.

^¶^ Aspirin‐sensitivity manifesting as urticaria.

BMI, body mass index; FODMAP, fermentable oligo‐, di‐, mono‐saccharides and polyols; IBS, irritable bowel syndrome; VAS, visual analogue scale.

Analysis of baseline food intake showed that the participant with known aspirin‐sensitivity was already unknowingly selecting low salicylate foods; this participant had low symptom scores at baseline. Another three participants had very low levels of current symptoms at baseline; these were consuming moderate amounts of salicylates. Four participants had mild to moderate symptoms during baseline and all were consuming high amounts of dietary salicylates. Most of the participants consumed moderate or high amounts of FODMAPs (Table 2). The diet during the washout period was similar to baseline (data not shown).

### Nutritional composition of interventional diets

The nutritional composition of the low and high‐salicylate diets as consumed by the participants is shown in Table 3. The only significant difference in nutrient content of the diets was the average daily intake of salicylates (mean 6.6 [range 5.5–7.8] g/day for low‐salicylate diet and 27.9 [20.8–33.7] g/day for the high‐salicylate diet [*P* < 0.001; paired *t* test]). The total FODMAP content was minimal and did not differ between the diets (2.30 *vs* 2.70 g/day, *P* > 0.05).

**Table 3:** The nutritional content of the interventional diets actually consumed by the 10 participants. Analyses were performed using FoodWorks 7 together with the Monash University database of fermentable oligo‐, di‐, mono‐saccharides and polyols (FODMAP) and salicylate food content and shown as mean (range). The only statistically significant difference between the diets was for total salicylate content (*P* < 0.001; paired *t* test)

	Low‐salicylate diet	High‐salicylate diet
Energy (MJ/day)	8503 (7318–9664)	8327 (7258–9842)
Protein (g/day)	121 (97–148)	103 (69–133)
Fat (g/day)	72 (29–112)	71 (26–115)
Carbohydrate (g/day)	212 (201–257)	218 (146–295)
Fiber (g/day)	29 (17–31)	27 (21–32)
FODMAPs† (g/day)		
Total oligosaccharides	1.06 (0.06–2.55)	1.05 (0.15–2.35)
Fructans	0.81 (0.04–2.15)	0.83 (0.13–0.98)
Galacto‐oligosaccharides	0.21 (0–0.39)	0.11 (0–0.41)
Excess fructose	0.89 (0–3.05)	0.61 (0–2.15)
Sorbitol	0.28 (0–0.62)	0.11 (0–0.26)
Mannitol	0.06 (0–0.28)	0.02 (0–0.04)
Lactose	0	0
Total salicylates (mg/day)	6.6 (5.5–7.8)	27.9 (20.8–33.7)
Gluten, preservatives, additives	Absent	Absent

^†^ FODMAP denotes fermentable oligo‐, di‐, mono‐saccharides and polyols.

### Symptoms

**Figure 1:**
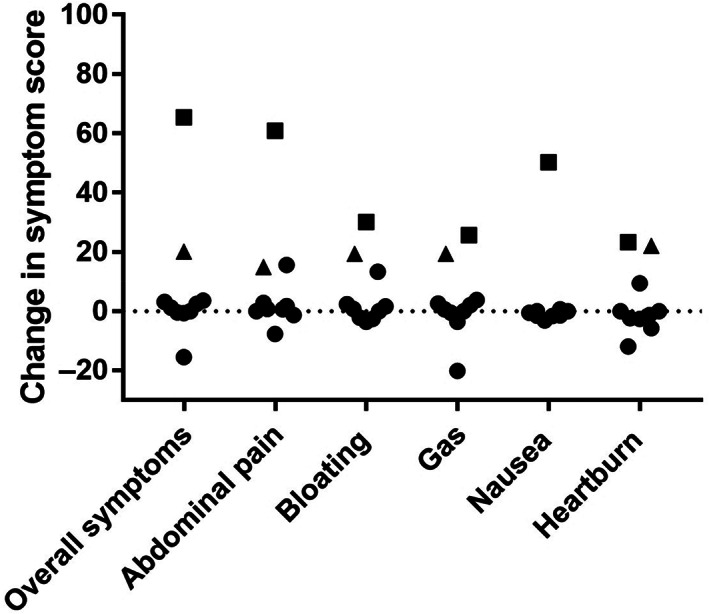
Difference in gastrointestinal symptoms between the low and high salicylates diets during the last 3 days of each dietary period according to the visual analogue scale. Results from the subject with aspirin‐sensitive urticaria are indicated by squares. The second subject who had increased symptoms with the high‐salicylate diet is indicated by the triangles.

Differences in symptoms during the last 3 days of the high‐ *versus* low‐salicylate diets are shown in Figure 1. For the entire cohort, no differences in overall symptom scores across the baseline, high‐salicylate diet, washout, and low‐salicylate diets were observed (*P* = 0.625; Friedman test). Likewise, there were no differences in the fecal symptoms (data not shown).

**Figure 2:**
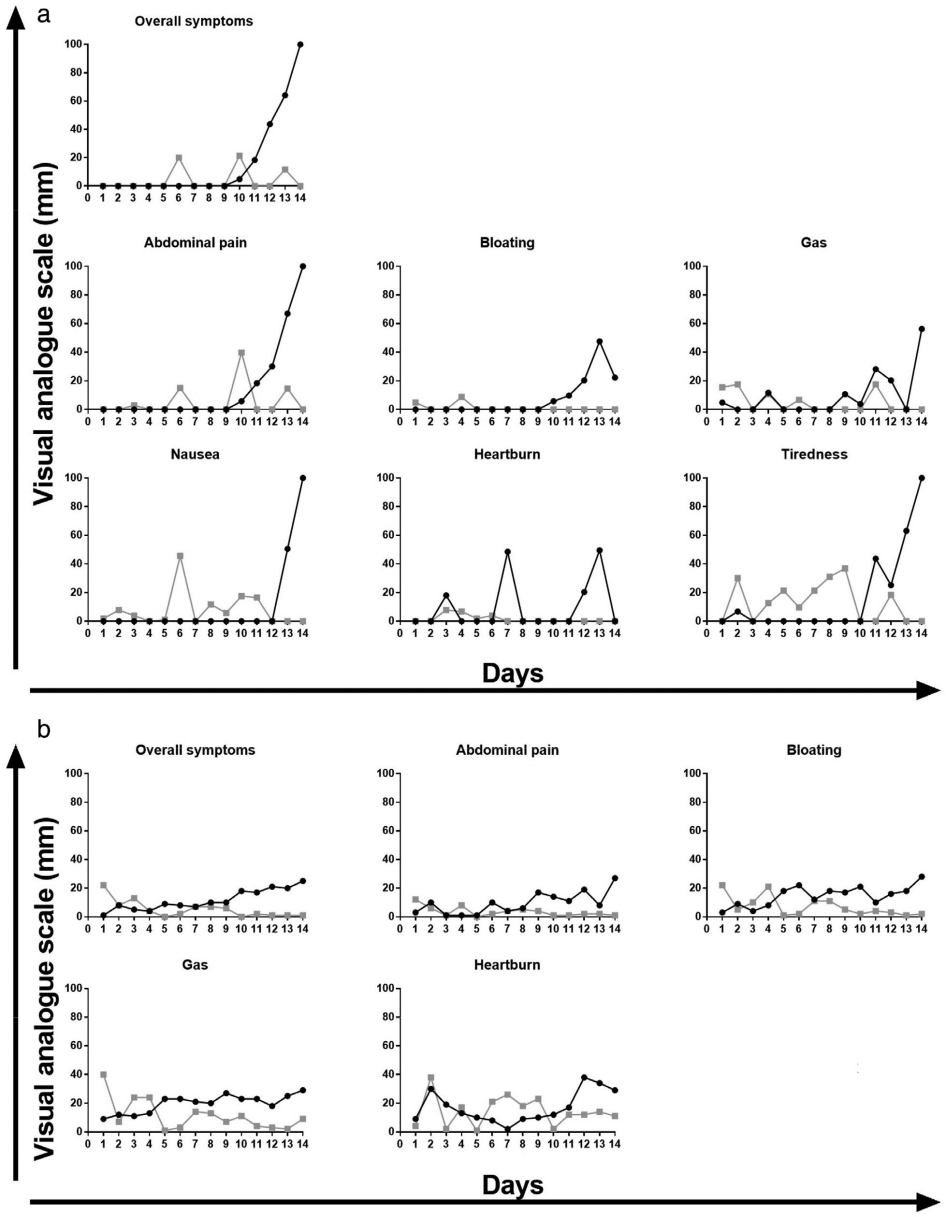
Daily symptom scores in the participants exhibiting consistent differences between the low‐ and high‐salicylate diets. (a) the subject with aspirin‐induced urticaria reported a marked increase in symptoms in the second week of the high‐salicylate diet. (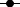), High salicylate diet; (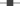), low salicylate diet. (b) the second subject showed steady reduction of symptoms with the low‐salicylate diet and an increase in abdominal symptoms in the second week of the high‐salicylate diet. She did not have nausea or tiredness. (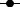), High salicylate diet; (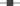), low salicylate diet.

Two participants had different levels of abdominal symptoms between the interventional diets. The aspirin‐sensitive participant showed worsening of all abdominal symptoms on the high‐salicylate diet (Fig. 1). The time‐course of symptom‐induction showed an increase in symptoms detected from day 9 onwards (Fig. 2a). Another participant with IBS but without known salicylate sensitivity also showed greater abdominal symptoms while consuming the high‐ compared with low‐salicylate diet, and this increase in symptom severity also occurred from day 9 (Fig. 2b). The baseline diet of this participant was rich in salicylates, and abdominal symptoms improved on the low‐salicylate diet compared with those during the baseline and washout periods (Fig. 2b). In the second week of the high‐salicylate diet, symptoms increased and reached levels observed at baseline.

No participants had asthma or a skin rash during the study. One participant complained of rhinitis and another mild headache, both during the low‐salicylate diet. Increased tiredness was reported by four participants, three on the low‐salicylate diet and one, the participant with aspirin‐sensitivity, during the high‐salicylate diet.

### Adherence and success of blinding

Dietary adherence was 100%, except one participant who was noncompliant to the low‐salicylate diet for 4 days and the high‐salicylate diet for 2 days. Qualitative feedback regarding the palatability of the diets was positive. Eight participants did not correctly identify the low‐ or high‐salicylate diets (including the aspirin‐sensitive participant).

## Discussion

While there seems little controversy that aspirin is associated with asthma and urticaria, the strength of data indicating that food‐related salicylates induces abdominal symptoms in patients with IBS is restricted to expert opinion based upon anecdotal evidence. In the current randomized, double‐blind, crossover trial, 2 of 10 participants had different symptom profiles associated with differences in salicylate intake. The first showed convincing moderate to severe symptoms on the high‐salicylate diet and few symptoms on the low‐salicylate diet. Interestingly, this participant habitually consumed a diet low in salicylates and had a previous diagnosis of aspirin‐induced urticaria, although skin lesions were not observed throughout the study. The other participant whose habitual diet was rich in salicylates improved on the low‐salicylate diet and reported worsening of symptoms when the high‐salicylate diet was introduced. Such findings suggest that sensitivity to food‐related salicylates exists but occurs uncommonly and with variable severity.

Other observations might support the hypothesis that these two participants were exhibiting salicylate sensitivity. First, both participants showed a pattern of slow induction/alleviation of symptoms over 1 week when exposed to differing amounts of dietary salicylates, consistent with the hypothesis that it is the accumulated tissue concentrations of salicylates that are associated with symptom genesis. According to this theory, the hypersensitive participants can tolerate a particular food chemical until it accumulates and crosses a tolerance threshold beyond which the symptoms manifest.^4^ Secondly, no evidence of a nocebo response was observed in the participants. When re‐challenging participants with self‐reported gluten sensitivity to blinded diets containing gluten or not, nocebo response is strong.^34^ Nine of the participants in the current study knew little of salicylate sensitivity and were not, therefore, anticipating severe symptoms as those who believe themselves sensitive to gluten might. The one participant who was aware of her aspirin sensitivity showed no symptomatic response to the low‐salicylate diet. This was not due to recognizing the nature of the diet because she unsuccessfully identified the diets at the end of the study.

From current dogma, it was anticipated that half of the participants would exhibit salicylate sensitivity. However, five of the participants were in relative remission from IBS symptoms at baseline, despite fulfilling the historical criteria for that diagnosis and their usual diets being moderate to high in salicylate content. In that setting, it would not be possible to show improvement by reducing salicylate intake. While it might also be argued the high‐salicylate diet would be unlikely to induce symptoms because they were already consuming moderate to high dietary salicylates, the diet provided had salicylate content (30 mg/day) greater than that likely to be sourced by the participants. Maximum daily intake has previously been estimated at 13 mg/day.^35^ In contrast, the other three participants without symptomatic changes to the altered dietary salicylate intake had symptoms at baseline and habitually consumed moderate to high amounts of salicylates in their usual diet.

One problem with dietary interventions is collinearity, whereby other changes to dietary composition might be causing or masking symptoms due to the targeted food component. One potential dietary confounder was FODMAP intake. Both experimental diets were, therefore, designed to be low in FODMAPs. Habitual intake indicated moderate intake of FODMAPs by most participants. Thus, lowering FODMAP intake may have masked induction of symptoms by salicylates, although the three participants who were moderately symptomatic did not improve despite the low FODMAP content of the interventional diets. Other dietary components with potential to induce symptoms (gluten, lactose, preservatives, and additives) were not used in the prepared diets. However, other natural food chemicals, namely glutamates and amines, were present in moderate levels in both diets. If these moderate levels represented a reduction in baseline intake for the participants, this reduction of putatively troublesome dietary factors may have masked symptom induction. However, no participant with baseline symptoms improved on both diets.

There have been few studies of dietary restriction in known salicylate‐sensitive conditions. The only cross‐over study reported compared 12 weeks of a low salicylate diet (0.01–0.09 mg salicylic acid/serve) compared with habitual diet in 10 patients with aspirin‐exacerbated respiratory disease.^36^ Symptoms of rhinosinusitis improved and inflammation in endoscopic sections of the sinuses reduced with the low‐salicylate diet. However, dietary salicylate restriction is not a routine therapy in aspirin‐associated conditions. The presence of functional gastrointestinal symptoms in patients with known salicylate‐sensitivity syndromes has been poorly documented. The observation that the salicylate‐sensitive participant in the current study chose foods low in salicylate content was not directed by her physician and presumably was the result of personal observations. Studies on the prevalence of gastrointestinal symptoms and on the effect of low‐salicylate diets in patients with known aspirin‐related syndromes are warranted.

The current pilot study has limitations. The small number studied, and their heterogeneity of baseline symptoms and salicylate intake limit the interpretation of the results. However, as a pilot, it did show that palatable diets differing in salicylate content can readily be created with its associated high compliance, that nocebo effects are unlikely where the participants studied have little prior knowledge of the putative dietary component, and that salicylate‐sensitive IBS symptoms may well occur if given for a period of greater than 1 week. While the study of patients who believed they were salicylate‐sensitive may have been more appropriate, this would likely to be associated with nocebo responses. Whether altered delivery of salicylates in the diets was achieved may have been enhanced by measurement of serum salicylate concentration at the end of each dietary period, but a very sensitive assay would be needed as only about 3% of dietary salicylate intake is bioavailable.^13^, ^37^

In conclusion, these results have provided some evidence that food‐related salicylates may influence the genesis and/or severity of functional gastrointestinal symptoms in patients with IBS. It will be important to extend this study into a larger cohort to determine the true incidence of salicylate sensitivity in IBS, identify potential markers to predict response, and elucidate mechanisms of action. The lack of discernible placebo and nocebo effects and the palatability of diets differing in salicylate content suggest that blinded cross‐over feeding studies may be a valid way of defining the sensitive population. The observation of a clear effect of varying dietary salicylates in a participant with aspirin‐mediated urticaria suggests that the use of dietary interventions in patients with known aspirin‐mediated medical conditions is worthy of further investigation.

## Supporting information


**Appendix** **S1.** Supporting Information.
